# Fibronectin as a multiregulatory molecule crucial in tumor matrisome: from structural and functional features to clinical practice in oncology

**DOI:** 10.1186/s13046-021-01908-8

**Published:** 2021-03-17

**Authors:** Sheila Spada, Annalisa Tocci, Francesca Di Modugno, Paola Nisticò

**Affiliations:** 1 Department of Radiation Oncology, Weill Cornell Medicine, New York, NY USA; 2 Tumor Immunology and Immunotherapy Unit, IRCCS-Regina Elena National Cancer Institute, Rome, Italy

**Keywords:** Fibronectin, Fibrillogenesis, Extracellular matrix, Actin cytoskeleton, Cancer-associated fibroblasts, Metastasis, Dormant cells, Metastatic niche, Extracellular vesicles, Cancer

## Abstract

Deciphering extracellular matrix (ECM) composition and architecture may represent a novel approach to identify diagnostic and therapeutic targets in cancer. Among the ECM components, fibronectin and its fibrillary assembly represent the scaffold to build up the entire ECM structure, deeply affecting its features. Herein we focus on this extraordinary protein starting from its complex structure and defining its role in cancer as prognostic and theranostic marker.

## Background

Tissue architecture is crucial for cell homeostasis and functions of each tissue and organ [1]. A multitude of biological activities essential for normal organ development and functions are tightly regulated by extracellular matrix (ECM) assembly, modification and degradation [2]. ECM composition is strictly related to the requirement of tissue functions and its composition is unique for each organ due to its crucial role for peculiar functions [3]. Dysregulation of ECM dynamics leads to various pathological conditions and is crucial in cancer development and progression [4].

Here we remark the fundamental role of fibronectin (FN1) as a key component of ECM and of its fibrillogenesis, a cytoskeleton contractility dependent process, which initiates the ECM elaboration and contributes to ECM architecture, responsible for the bioavailability of growth factors and cytokines. We discuss the involvement of fibroblasts and in particular of cancer-associated fibroblasts (CAFs) in the FN1 production and assembly and the effects in tumor progression, invasion and metastasis with a focus on the role of extracellular vesicles (EVs) in FN1-driven signaling. Finally, the potential employment of FN1 as a useful biomarker and valuable target for clinical applications, including cancer diagnosis and therapy is debated.

### Fibronectin gene description, splicing, functions

FN1 is one of the most abundant and ubiquitous glycoprotein in the ECM, which has diverse biological roles in development, cellular growth and differentiation, adhesion, migration [5–8], and wound healing [9, 10] mainly through integrin-mediated signaling. This crucial role in a physiological microenvironment is even more significant in tumors where the reprogramming of the stroma is accompanied by an up-regulation of ECM proteins and their receptors, with FN1 as an important component of the tumor matrisome [4, 11, 12].

**Fig. 1:**
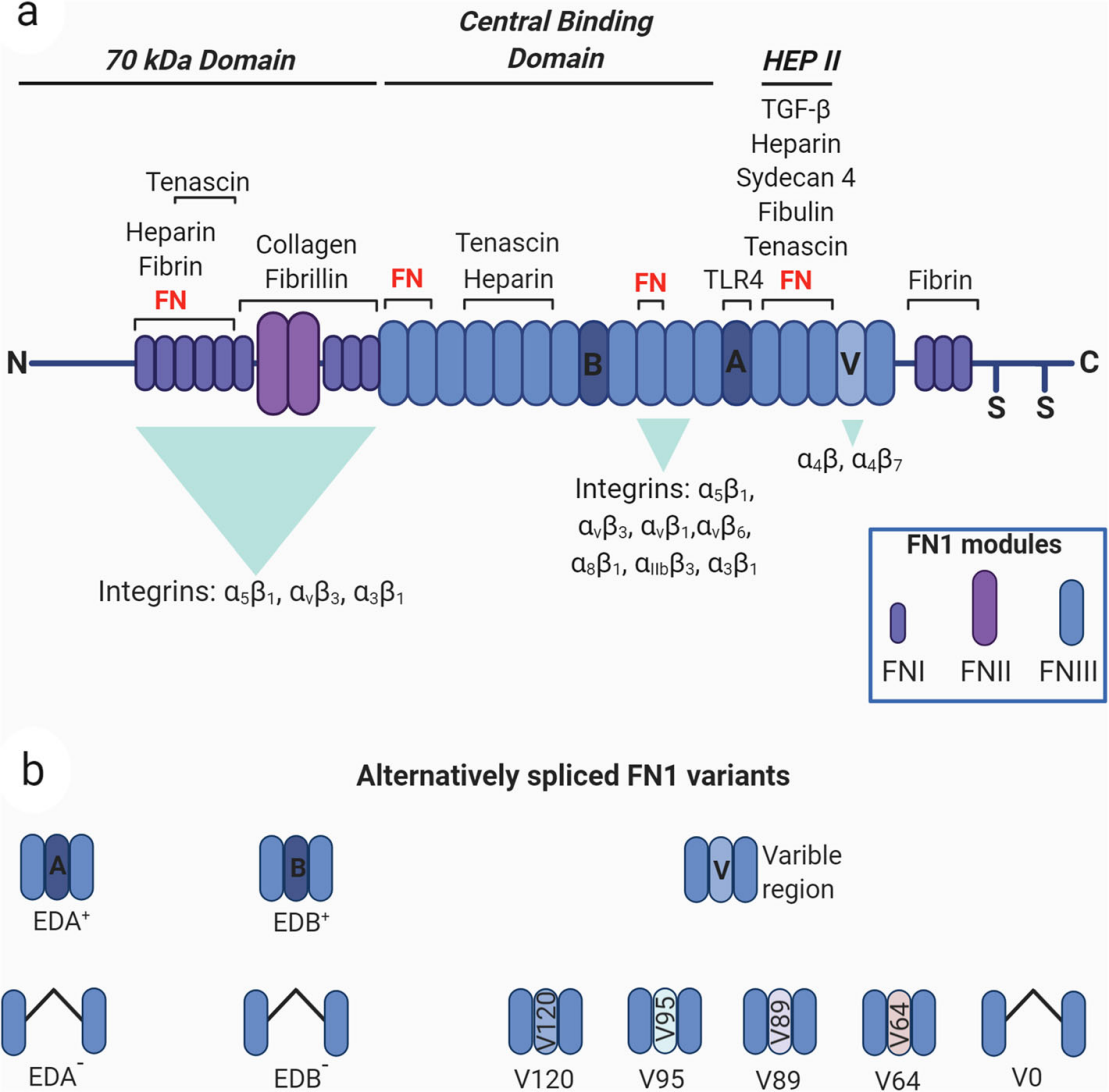
Diagram of FN1 structure and its splice variants. a) FN1 monomer is composed by type I, type II and type III modules (FN I-III). The type III EDA (**a**) and EDB (**b**) repeats and the variable region V are alternatively spliced. The binding repeats for cellular surface integrin receptors and for other proteins, such as FN1, heparin, fibrin, collagen, fibrillin, tenascin, TGFβ, syndecan 4 and fibulin, are reported. The modules are grouped into functional domains: N-terminal 70-kDa domain (FNI1–9), the 120-kDa central binding domain (FNIII1–12) and the heparin-binding domain HEPII (FNIII12–14). The two cysteine residues at the C-terminus possess the thiol functional groups to build disulfide bonds with another FN1 monomer forming the FN1 dimeric protein. b) Schematic representation of FN1 alternative splicing variants. Created with BioRender.com

FN1 is a highly conserved protein composed of two similar but not identical monomers of 220 and 250 kDa linked by two disulfide bridges at the C terminus of the protein [6, 13]*.* Each monomer is composed by three distinct modules termed as type I (FNI), type II (FNII) and type III (FNIII) modules [14]. FN1 contains 12 FNI repeats, 2 FNII repeats, 15 constitutively expressed and 2 alternatively spliced (termed Extra Domains, EDA and EDB) FNIII repeats and a non-homologous variable (V) or type III connecting segment region (IIICS) [15, 16]. FNI modules are composed by 45 amino acids, FNII modules by 60 amino acids, which contain cysteine residues responsible for the formation of intra-domain disulphide bridge [17, 18] (Fig. 1a).

The largest part of FN1 structure is constituted by FNIII modules, each formed by a consensus sequence of about 90 residues organised in seven β strands which form two antiparallel β sheets [19].

From a single 75-kb gene located on chromosome 2 and composed of 47 exons, 20 different isoforms are generated through alternative splicing [20, 21] enabling FN1 to exert different biological functions by interacting with ECM components and a huge number of integrin receptors. The inclusion of the alternative splicing regions is elevated during embryonic development, whereas reduced after birth and with aging [22, 23]. During adult life an intense splicing activity of the FN1 gene occurs in angiogenesis, tissue repair, fibrosis and of relevance in cancer where FN1 modifications strongly contribute to the age-related alterations in the ECM biosynthesis and degradation [24], thus modulating the tumor microenvironment (TME) composition and cancer progression [25, 26].

FN1 exists soluble as a dimer in the plasma (plasma fibronectin, pFN1) and as an insoluble part of a fibrillar network in the ECM (cellular fibronectin, cFN). Plasma FN1 is produced and secreted by hepatocytes directly into the blood stream in a soluble and inactive heterodimeric form [27] containing one or two IIICS segments in each subunit but lacking the EDB and EDA modules [28].

Cellular FN1 consists of a heterogeneous group of isoforms, constituted by variable proportion of the EDA and EDB domains and of the IIICS (Fig. 1b), which participate in ECM composition in a tissue-specific manner. It is produced by a variety of cell types including endothelial cells, chondrocytes, synovial cells and myocytes, but mainly by fibroblasts [29]. The diversity of functional domains allows FN1 to have many binding partners such as collagen [30], fibrin [31, 32], heparin [33], a variety of cell receptors [15] and FN1 itself [34–37]. This complex structure allows FN1 to accomplish various biological [35] roles and to dynamically respond to the changes of the environment.

The EDA domain is involved in the regulation of multiple biological functions [38–42] as shown in mice lacking EDA regulated splicing, which present abnormal skin wound healing and a shorter life compared to control mice [43]. The role of EDA in pathological processes such as cancer has been demonstrated by the work of Manabe and colleagues who revealed the importance of EDA+ FN1 in promoting cell cycle progression through the induction of cyclin D1 expression, hyperphosphorylation of pRb, and activation of mitogen-activated protein kinase extracellular signal regulated kinase 2 (ERK2) [44]. Moreover, cFN containing EDA domain, produced by endothelial cells, induces Epithelial to Mesenchymal Transition (EMT) in colorectal cancer cells and plays a pivotal role in promoting colorectal cancer metastasis [45]. During lung fibrosis, transforming growth factor- β (TGF-β) regulates the inclusion of the EDA exon in mature mRNA coding for cFN [46, 47]. In turn, the presence of EDA+ cFN is required for the activation of latent TGF-β [48] and TGF-β1 together with EDA+ cFN drive the activation of fibroblasts into α-*smooth muscle actin* (α-SMA) expressing myofibroblasts [39].

Furthermore, EDA domain can trigger the inflammatory response trough the binding and the activation of Toll Like Receptor 4 (TLR4) [42]. It has been shown that the activation of TLR4 by EDA+ FN in mesenchymal cells leads to a pro-fibrotic gene program characterized by the up-regulation of genes involved in wound healing, tissue repair and ECM remodelling [49, 50].

Less is known about the function of the EDB domain. EDB deficient mice develop normally and are fertile [51]. Interestingly EDB+ FN is rarely found in healthy adults, while it is highly expressed in tumour vasculature [52]. The absence of EDB+ FN in healthy tissue and its specific presence in tumour, makes this domain a promising candidate for the development of therapeutic agents as well as a biomarker in various types of cancer [53, 54].

By the use of biologically active recombinant EDB+ cFN it was observed that EDB+ cFN is incorporated more efficiently into the ECM [55] and EDB^−/−^ embryonic fibroblasts (MEFs) grow slowly and produce thinner and shorter fibrils compared to control MEFs, indicating a role of this domain in the efficacious assembly of FN1 ECM [51].

### FN1 extracellular matrix assembly

**Fig. 2:**
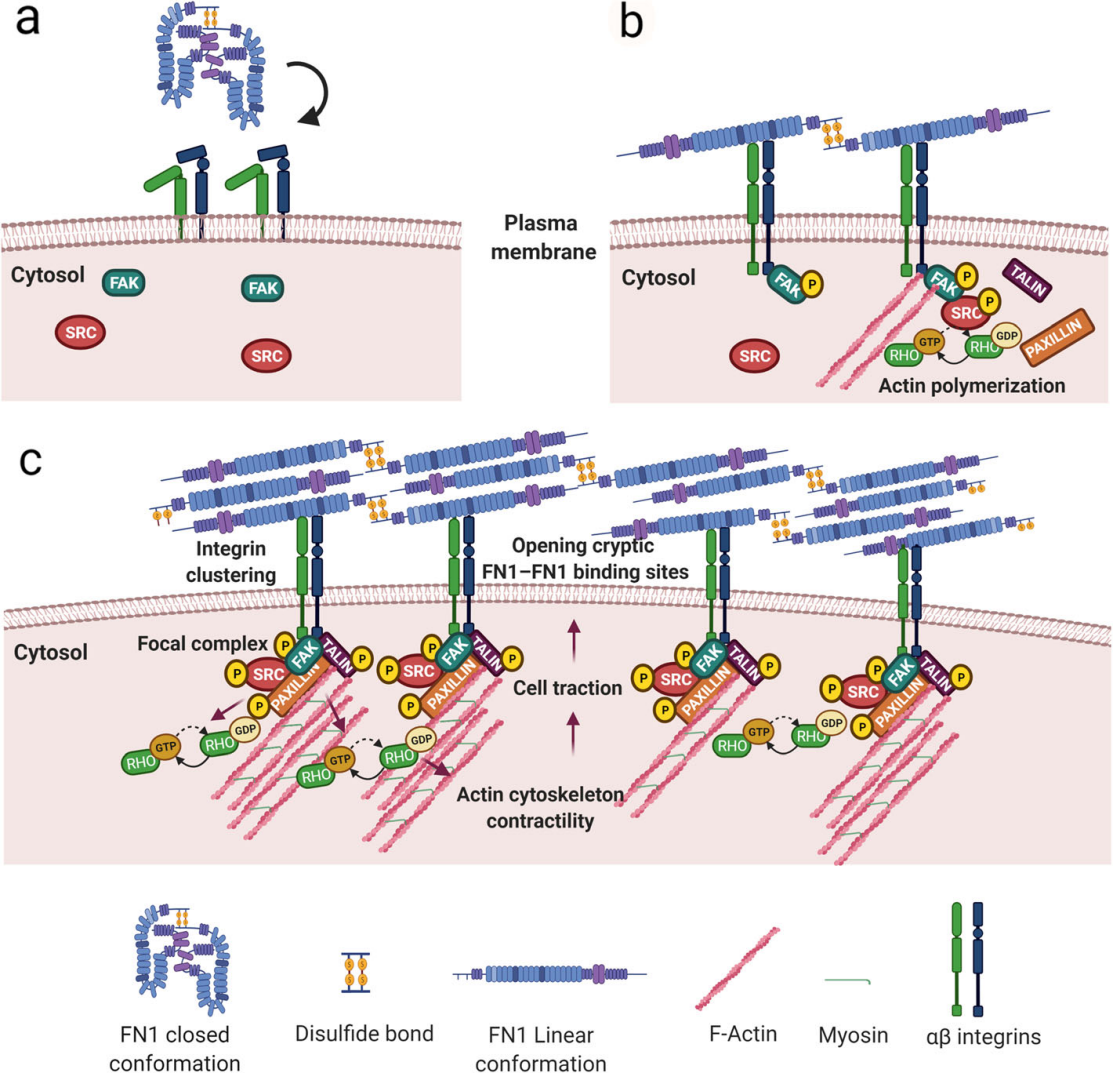
FN1 fibrillogenesis process. **a** FN1 dimers have a compact conformation mediated by disulfide bonds at the C terminus of each subunit. **b** The FN1 binding to integrins induces conformational changes unfolding the protein, bring closer FN1 molecules at the cell surface and determines integrin clustering and activation with the recruitment of focal adhesion kinase (FAK), Src kinase, paxillin, and talin to generate focal complexes that activate polymerization of actin filaments. **c** The actin cytoskeletal network associates with the cytoplasmic domains of integrins forming a connection that is crucial for initial FN1 matrix generation. The RhoA GTPase-mediated cytoskeleton contractility and actin–myosin interactions induce cell traction, generating FN1 conformational alterations, thus opening cryptic FN1–FN1 binding sites, a requisite for FN1 fibrillogenesis. Created with BioRender.com

The role of FN1 in ECM composition during development, in tissue homeostasis and cancer is mainly attributable to cFN1. Dimers of cellular FN1 have intrinsic and peculiar properties fundamental in orchestrating vital processes, including the elaboration of ECM within tissues and organs. This resides in its ability to generate fibrillar FN1 matrix, a cell-dependent [56, 57] dynamic and complex process that involves the multimodular and multidomain structure of the FN1 [58–60] (Fig. 2).

#### FN1 fibrillogenesis and the role of actin cytoskeleton contractility

The FN1 fibril formation is a multistep process which consists of the association of multiple FN1 molecules and starts with the binding of secreted FN1 dimers to cell surface integrins. The α5β1 integrin is the primary receptor mediating the assembly process [61], as demonstrated by blocking antibodies against anti-integrin or anti-FN1 [61, 62]. FN1 binds α5β1 integrin via the integrin-recognition sequences being the RGD sequence in the module III10 the most explored [63]. A further binding to the synergy site in III9 promotes the specificity of α5β1 [64–66]. However, other integrins could mediate FN1 fibril formation and other receptors such as syndecans have been implicated in the FN1-integrin interaction process. Syndecan-4 glycosaminoglycan chains bind the HepII domain of FN1 in the proximity of α5β1 integrin-binding domain, and is included into nascent adhesions, suggesting that it participates in cell-FN1 interaction [67]. Similarly, syndecan-2 is needed for FN1 matrix assembly [68].

When in solution FN1 dimers have a compact conformation mediated by both interchain disulfide bonds at the C terminus of each subunit and intramolecular disulfide bonds within each type I and type II module [37, 57, 69, 70] (Fig. 2a). The FN1 binding to integrins induces receptor clustering which brings closer many FN1 molecules at the cell surface and determines integrin activation with the recruitment of focal adhesion kinase (FAK), Src kinase, paxillin, and talin to generate focal complexes that activate polymerization of actin filaments [57, 71] (Fig. 2b and c). The actin cytoskeletal network associates with the cytoplasmic domains of integrins forming a connection that is crucial for initial FN1 matrix generation [72]. The RhoA GTPase-mediated cytoskeleton contractility and actin–myosin interactions induce cell traction generating FN1 conformational alterations, thus opening cryptic FN1–FN1 binding sites, a requisite for FN1 fibrillogenesis [73–76] (Fig. 1c). Indeed, the inhibition of fibril formation has been reported after the integrin cytoplasmic β domain deletion, treatment with actin depolymerization drugs [72] and RhoA GTPase inhibition [75]. The relevance of actin dynamics and actin binding proteins in fibrillogenesis is also underlined by the finding that the actin regulator Mena, a member of Ena/VASP family, is able to bind the cytoplasmic tail of α5 integrin and to promote FN1 fibrillogenesis in murine fibroblasts [77].

The cryptic FN1 sites reside within the FNIII modules [78] which, upon mechanical cell traction, expose cryptic binding sites allowing FN matrix assembly, as demonstrated by antibodies that recognize this region [79, 80] and by specific FN1 deletions, indicating that the III2 repeat is a critical module for fibrils formation [81]. The type III modules contain numerous binding sites for the amino-terminal assembly domain, a fragment of 70-kDa, which represents the principal site for FN1 self-association, as demonstrated by deletion, mutagenesis, and antibody-blocking studies [62, 82–84]. During the initial phases of FN1–FN1 interactions, short fibers accumulate at the cell surface, which become longer with the consecutive FN1 dimers inclusion. At this point, FN1 dimers could also become associated to growing fibrils independently of integrin binding [85] and as the matrix assembly process proceeds the fibrils are converted into a deoxycholate detergent (DOC) insoluble form, an irreversible process [86] which has been utilized to experimentally distinguish the amount of soluble and fibrillar FN1.

Besides the DOC solubility, the fibrillogenesis process has been monitored by immunofluorescence staining and FN1 was the first fluorescence resonance energy transfer (FRET) tension sensor that has been generated [87]. FRET experimental data demonstrated that forces generated by cells and controlled by cytoskeletal tension, induces a stretching and thus a mechanical unfolding of the FN1, enabling its conversion from solution to fibrils [76], and paved the way to explore the growing field of mechanobiology in live-cell imaging [88]. The FN1 matrix assembly provides the ground for the deposition of others ECM components such as type I collagen and thrombospondin-1 [89]. In fact, collagen peptides bind to different sites in the modules I of FN1 [90] and in the absence of FN1 in cell culture collagen I is not organized into fibrils [91–93]. Contextually to collagen, FN1 fibrils are also associated with fibrillin [94], fibulin [95], tenascin-C and elastin [96]. Interestingly, lysyl oxidase (LOX), the enzyme that catalyzes the covalent cross-linking of fibrillar collagens and elastin, binds to the cellular form of FN1, and this interaction regulates LOX catalytic activity [97]. These data suggest that FN1 fibrillogenesis represents a first step for the development of a functional and organized ECM, generating not only a structural support for the tissues, but also providing biomechanical signaling in both physiological or pathological conditions [98]. Noteworthy, ECM and in particular FN1-rich ECM is a reservoir for growth factors and cytokines, including members of the platelet-derived growth factor and fibroblast growth factor families [99], vascular endothelial growth factors (VEGFs) [100, 101], the tumor necrosis factor α (TNF-α) [102] and the latent TGF-β1complex [103–105], providing the spatial and temporal definition of their availability and activity [99, 100, 106–108].

#### FN1 fibrillogenesis and TGFβ cross-regulation

TGF-β importantly affects several cellular processes and has been implicated in the EMT process affecting the progression of various solid tumors [109]. TGF-β, produced by cells in a latent form, links to the ECM via the binding with the latent TGFβ-binding protein 1 (LTBP-1) that associates with FN1 fibrils and fibrillin microfibrils [110, 111]. Massam-Wu and coauthors have demonstrated that the TGF-β bioavailability is controlled by the deposition of a large latent TGF-β complex in dependence of the pericellular assembly of fibrillin microfibrils, which interact with FN1 during fibrillogenesis [112]. On the other hand, Griggs and coauthors showed that exogenous active TGF-β1 initiates the EMT process in MCF10 breast cells by upregulating the expression and secretion of FN1 and the TGF-β1 complex. Reciprocally, the LTBP-1/latent TGF-β1 complex localization to assembled FN1 fibrils is necessary for complete EMT [113]. Furthermore, TGF-β can regulate the ECM by inducing FN1 synthesis and its incorporation into ECM [114]. These data demonstrate that, although the cross-talk between FN1 expression and fibrillogenesis and TGFβ signaling is complex, the two pathways are interconnected and reciprocally regulated. In this direction, Varadaraj and coauthors revealed stable interactions between the cytoplasmic domain of the type II TGF-β receptor (TβRII) and the FN1 receptor (α5β1 integrin) and found that, in response to TGF-β, cell surface–internalized FN1 is not degraded by the lysosomes but instead undergoes recycling and incorporation into fibrils, a process dependent on TβRII [115]. This elegant work describes a non- transcriptional source of fibrillogenesis which, as the Authors suggest, may exert a crucial role in rapid ECM remodeling and sustained growth factor signaling, with implications for pathological disorders, such as fibrosis and cancer.

#### FN1 fibrillogenesis, a job of fibroblasts and CAFs

Fibroblasts are the major producers of ECM in normal development and tissue homeostasis and are crucial in communicating with many cell types [116, 117]. After tissue injury, fibroblasts produce different cytokines and growth factors, including TGFβ and differentiate into highly contractile phenotype characterized by α-SMA expression, termed myofibroblasts [118]. Myofibroblasts are implicated in the abnormal accumulation and excessive remodeling of ECM that characterize fibrosis [119, 120], a destructive disease, which induces increased tissue stiffness and loss of organ function, especially in lung [121]. Myofibroblast activation occurs in response to various stimuli including active TGF-β1 [122] and EDA-containing fibronectin [39]. When fibrosis is in advanced stage, the high ECM stiffness induces fibroblasts to co-express EDA+ FN and LTBP-1 favoring their interaction in the ECM, suggesting that the inhibition of LTBP-1 and EDA+ FN interaction may reduce the TGF-β1 reservoir in the ECM [123] and fibrosis. The interference with the contractile function of myofibroblasts may be another potential therapeutic option for fibrosis, as suggested by Torr and coauthors who have differentiated human pulmonary fibroblasts in myofibroblasts by TGF-β1 treatment and then inhibited fibrillogenesis by pharmacologic disruption of the transcription factor megakaryoblastic leukemia-1 (MKL1)/serum response factor (SRF), or by of the depletion of MKL1/SRF target gene, the α-SMA [124].

Cancer-associated fibroblasts (CAFs) can affect multiple aspects of tumor progression either by secreting soluble stimuli or by remodeling the ECM [116, 125–127]. Different CAFs subtypes have been reported with different functions in cancer [128–131] and recently, by single cell RNA-Seq analysis, a cluster of CAFs characterized by high expression of genes coding extracellular matrix, ecm-myCAF, has been identified and associated with an immunosuppressive properties [132].

The ecm-myCAF subtype overexpress hMENA, that we have recently demonstrated as crucial player in pro-tumor CAF activation and in cancer cell-CAF crosstalk [133].

FN1 assembly has been suggested as a new hallmark of CAFs that promote tumor invasion as indicated in colon cancer-derived CAFs, where CAF contractility induces FN1 assembly and tumor cell invasion [134]. Similarly, in prostate cancer it has been shown that CAF-mediated FN1 fibrillogenesis promotes CAF–cancer cell interactions and guides cancer cell migration. The Authors also found that CAF-derived matrices, but not matrices derived from normal fibroblasts, exhibit aligned fiber organization and promote directional cancer cell migration, due to enhanced contractility α5β1 integrin-ECM-mediated [135]. Besides CAFs, also tumor cells have been implicated in the fibrillogenesis process affecting tumor cell survival. Recently Sahai and coauthors elegantly demonstrated that breast cancer cells metastasizing into the lung are induced to generate FN1 fibrils by their interaction with resident alveolar epithelial cells. This in turn promotes the generation of integrin-dependent pro-survival signals mediated by FN1 fibrillogenesis and enables the survival of indolent metastasizing cells [136]. Collectively, these data indicate that the process responsible for FN1 matrix assembly is highly influenced by different cells and cues in the microenvironment.

### FN1 in tumor progression, invasion and pre-metastatic and metastatic disease

**Fig. 3:**
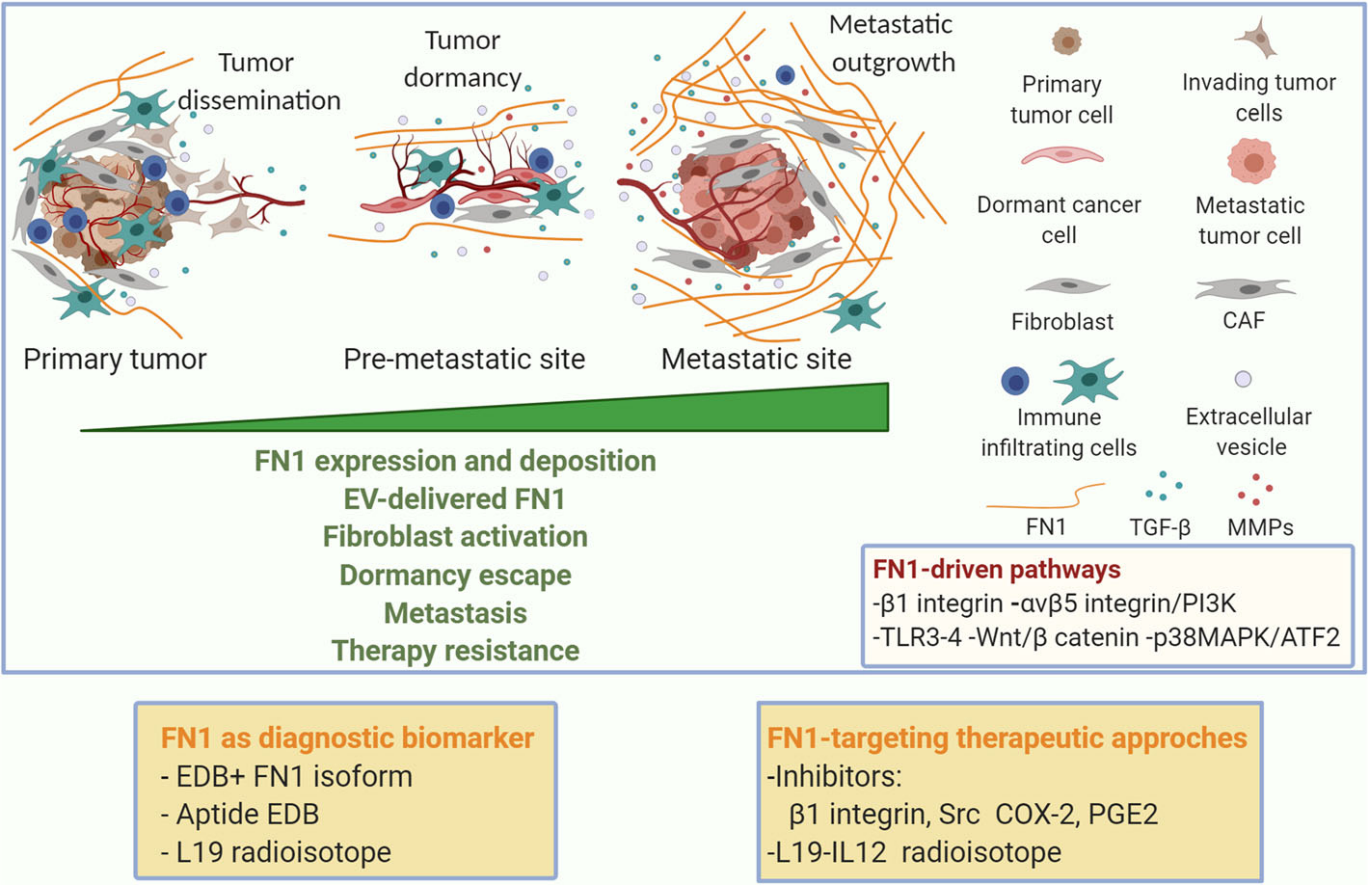
FN1 as player in metastatic process and as diagnostic and therapeutic target. At the primary tumor site, the cancer cells and cancer associated fibroblasts (CAFs) deposit FN1. FN1 activates intracellular signaling, mediated by integrins, TLRs, Wnt/β catenin, and P13K, that lead to an increase of expression and secretion of FN1, MMPs, TGFβ. In parallel, FN1 is transferred by EVs. Cancer cells, with invasive capabilities, extravasate and reach a secondary organ, where tumor dormancy immune controlled may occur. Dormancy escape arises in the metastatic outgrowth in a permissive microenvironment with FN1 and collagen enriched ECM, affecting resistance to therapy. EDB isoform of FN1 is used as a biomarker for cancer diagnostic imaging and FN1 pathway as a target for therapeutic applications. Created with BioRender.com

The cross-talk between tumor cells and the non-cellular and cellular microenvironment components, including non-tumoral resident and immune cells, influences the tumor progression [137]. A late step of the tumor progression is the metastasis, which can shortly occur after primary tumor diagnosis or years later, sometimes after decades. Late relapses have been ascribed to the tumor metastatic dormancy, which refers to the activation of a cell quiescence program [138] and the failure of tumor cells to engage signals for adhesion via integrin β1 hampering the activation of pro-proliferative signaling pathways [139] i.e. ERK [140]. Furthermore, dormancy has been also identified as a key feature of cells resistant to cancer drugs [139]. Upon extravasation, the cancer cells colonize the target organ encountering a new ECM produced from local stromal cells and start receiving signals that determine the fate of the metastatic cells in the new place. The cells could remain in a dormant state [141] maintaining a balance between proliferation and apoptosis keeping constant the cell number with an undetectable size [142]. Once in the second organ cells communicate with a new ECM which is critical in maintaining or outbreaking dormancy [143] (Fig. 3). Among the different ECM components, FN1 has been reported as a main driver of tumor progression by different mechanisms including the increase of growth factors signaling via integrin clustering [144, 145] (Fig. 3). For instance, FN1 is upregulated in the metastatic niche when lung as well as melanoma cell lines are implanted in lung [146]. The resident stromal cells, activated by tumor cells, produce FN1 which sustains the recruitment of hematopoietic bone marrow progenitor cells which express the FN1 receptor α4β1 integrin, establishing the basis for a nascent metastatic niche [146]. Fibrotic lung-derived fibroblasts produce high level of FN1 and the secreted phosphoprotein 1 (SPP1) that chemoattract tumor cells and inhibit their apoptosis by binding to the common integrin αv receptor, expressed by tumor cells. These events promote metastatic seeding and outgrowth of the tumor cells in the fibrotic lung [147]. Additionally, α5β1 integrin with the down-stream JNK/c-JUN signaling regulates the matrix stiffness that induces LOXL2 upregulation which in turn leads to FN1 production, metalloproteinase 9 (MMP-9) and CXCL2 expression as well as bone marrow-derived dendritic cell recruitment. Altogether these events promote the pre-metastatic niche formation in hepatocellular carcinoma [148].

Similarly, lung alveolar epithelial cells have been reported to induce Src-mediated Secreted frizzled-related protein 2 (SFRP2) in breast cancer cells promoting FN1 fibril formation and cancer cell survival [136]. Although the mechanism by which SFRP2 induces FN1 fibril formation is still not elucidated, Montagner and coauthors suggest that insoluble extracellular SFRP2 promotes cell numbers by increasing the deposition and organization of fibronectin and activates the survival pathway upon integrin-FN1 binding (136). Interestingly, Barkan and colleagues demonstrated the role of FN1 in awakening dormant cells to a proliferative state, showing that FN1 binding to integrin β1 triggers downstream signaling which results in filamentous actin stress fiber formation and cell proliferation in the lung [149]. Similarly, our group has recently reported that the two alternatively expressed isoforms of the actin regulator hMENA (the anti-invasive hMENA^11a^ and the pro-invasive hMENAΔv6) affect the activation of β1 integrin and its downstream signaling in an opposite manner in cancer cell lines and the expression of FN1 also in the primary lung tumor tissues. These data have a strong clinical implication since in primary tumors high hMENA^11a^ correlates with low stromal FN1 and a favorable clinical outcome of early node-negative non-small-cell lung cancer (NSCLC) patients, providing a new tool for the stratification of patient risk, guiding their clinical management [150]. Exogenous FN1 added to NSCLC cells induces the phosphorylation of the upstream mTOR regulator AKT, reduces PTEN expression level in a time-dependent and dose-dependent manner, and inhibits the expression of two downstream mTOR regulators, LKB1 and AMPKα. Overall these events promote the activation of PI3K/Akt pathway downstream to α5β1 integrin with consequent NSCLC cell proliferation [151]. Moreover, FN1 also induces cell proliferation regulating cancerous inhibitor of protein phosphatase 2A (CIP2A) expression via its stabilization mediated by β catenin in bladder cancer [152]. However, the recipient ECM may also provide signals able to prevent dormant cell reactivation, as reported by Barney et al. who showed that TGF-β-mediated secretion of FN1 in dormant cells induces a well-organized and assembled dense FN1 matrix that contributes to maintain a dormant state. FN1 degradation due to MMP-2 disassembly occurred during the transition to outgrowth and let the dormant cells to enter into the cell cycle [153]. On the other hand, in pancreatic cancer plasma FN1 acts as a molecular switch, affecting the activity of the matricellular protein, SPARC and controlling whether SPARC promotes pancreatic cancer cell proliferation or induces cancer cell death. The Authors showed that inhibiting the interaction between SPARC and fibronectin prevents SPARC from inducing tumour growth and induces its pro-apoptotic effect on pancreatic cancer cell, by inducing Caspase-3/7-activity [154]. Further investigation to decipher microenvironmental cues favoring FN1-integrin pathways are needed to identify key signaling components that participate to dormant cell reactivation, offering new targets and therapeutic agents to fight metastatic disease.

#### FN1 and extracellular vesicles: potential biomarkers of invasion and metastasis

Numerous evidence describes the relevance of EVs as mediators of biological information with a great impact in cancer [155, 156]. EVs are nano-extracellular vesicles mainly distinguished in exosomes and microvesicles by their size, biogenesis and protein profile [157–159]. They are secreted by healthy as well as diseased cells, including cancer cells. EVs carry biologically active macromolecules, including DNA, RNA, proteins, lipids, and represent an efficient and powerful system of cell-cell communication with impact in physiological and pathological conditions [160]. Nowadays, EVs further represent an ideal tool for diagnosis and as biomarker to monitor the response to cancer treatments.

In this section, we will discuss FN1 delivery by EVs, the role of EVs in FN1-driven signaling and the potential use of FN1 included in EVs as a cancer biomarker.

EVs released by the tumor cells affect functionality and activity of different cell components of the cancer microenvironment and may promote invasion and metastasis [143, 161–164], as shown for exosomes derived from primary lung cancer cells which induce upregulation of genes involved in cancer cell invasion and migration to metastatic niche [143]. In vivo administration of tumor-derived exosomes in murine lung increases the expression of genes coding for ECM components, including FN1 and MMP-9, as well as proinflammatory and prometastatic chemokines [164]. Tumor exosomal RNAs participate in the pre-metastatic niche formation in the lung and induce neutrophil recruitment via-toll-like receptor 3 (TLR3) activation on alveolar epithelial type 2 cells, as demonstrated in TLR3 −/− mice. Indeed, in the absence of TLR3, FN1 and the other upregulated genes, neutrophil recruitment and lung metastasis are reduced [164]. Moreover, EVs derived from primary tumors with lung tropism carrying FN1 to recipient cells, contribute to establish a permissive pro-metastatic microenvironment as shown by Deng and colleagues. They showed that FN1 enclosed into or absorbed on the surface of exosomes derived from breast cancer cells co-cultured with tumor-infiltrating leukocytes, favors exosome-dependent cancer cell invasion and tumor metastasis in the lung [165]. Notably, EVs also participate at ECM remodeling that occurs during cancer progression. Exosomes derived from breast cancer cells target the lung fibroblasts and, upon their uptake, fibroblasts switch to an activated phenotype with FN1 deposition, ECM remodeling and pre-metastatic niche formation [166]. FN1 enriched exosomes advantage the invasive capability of fibroblasts, a crucial event in lung fibrosis. Indeed, Chanda et al. reported that fibroblasts shed EVs which express FN1 on their surface that in turn recruits lung fibroblasts and mediates their migration and invasion. The mechanism underlying this induction is related to the interaction between the exosomal FN1 and a5β1 integrin expressed by the recipient cells. FN1- α5β1 complex results in the activation of downstream kinases FAK and Src and ending up in the EVs-driven fibroblast invasion [167]. Recently, it has been shown that Tissue transglutaminase 2, an enzyme that crosslinks FN1 onto EVs, participates to the EV-mediated lung fibroblast reprogramming, contributing, along with fibrillar FN1 to metastatic niche development [168].

Based on the data above reported EVs may resemble the parent cancer cell assets, thus emerging as potential diagnostic and prognostic biomarkers. Finding out new potential biomarkers, An and colleagues analyzed the proteomic profile of EVs isolated from early and advanced-stage NSCLC patient sera and lung adenocarcinoma A549 cell line, compared to healthy donor sera and bronchial epithelial cell lines, respectively. The results revealed that among differentially expressed proteins, FN1 is over-expressed on cancer cells-derived EVs and may be a suitable, specific and sensitive diagnostic biomarker for NSCLC [169]. In conclusion, considering that EVs are stable in the blood representing a non-invasive source for monitoring cancer disease, the FN1-enriched EVs may represent new prognostic and theranostic markers in the clinical management of cancer.

### FN1: a potential biomarker in clinical cancer management

Changes in FN1 expression level, organization or degradation are related to several types of cancer [29, 170, 171], such as lung [172], breast [173, 174], prostate [175], bladder [176], head and neck [177] and colorectal cancer [178], candidating FN1 as biomarker for diagnosis or monitoring cancer progression.

FN1 soluble into the plasma has been proposed as a promising tool for cancer screening based on preclinical and clinical data. Plasmatic FN1 promotes lung metastasis in a murine model and contributes to fibrin clot formation and cancer invasion by activating αvβ3 integrin [179]. FN1 deposits could be detected in vessels of human metastatic livers and represent the substrate for circulating tumor cells to adhere to endothelial cells, to extravasate and form metastasis [180].

The limited expression of EDB+ FN1 isoform in normal adult tissue and its expression in tumor and vascular endothelial cells render this isoform a good biomarker not only as parameter of cancer angiogenesis but also as target for cancer imaging. Cancer diagnostic imaging has undergone continuous innovation allowing accurate characterization and monitoring of neoplastic disease [181] and multiple FN1 ligand-agents have been developed for molecular imaging, including nuclear magnetic resonance (MR) and fluorescence imaging. The antibody L19 labeled with γ-emitter ^123^I that targets EDB is used to localize the lesion in lung cancer as well as in brain and colorectal cancer with immunoscintigraphy [182]. The L19 has also therapeutic effect when fused with interleukin-12 (IL-12) increasing its anti-metastatic effect in colon adenocarcinoma [183]. The aptide EDB-conjugated superparamagnetic iron oxide nanoparticles detect lung carcinoma which overexpress EDB in vivo by MRI [183].

Therefore, FN1 is a potentially useful biomarker and target for several clinical applications, including cancer diagnosis and therapy.

### FN1 in cancer therapy and resistance

Accumulating evidence shows that pathways related to FN1 axis are promising therapeutic targets in cancer treatment [181]. Different strategies have been pursued to target FN1 and its integrin receptors, as shown by the use of COX-2 inhibitors in models of tobacco-driven lung cancer. These drugs inhibit FN1-dependent survival signals mediated by COX-2 and PGE2 upregulation [184]. Moreover, in human lung cancer cells, the FN1 gene expression is suppressed by thiazolidinediones (TZDs), ligands of the peroxisome proliferator-activated receptors-γ (PPAR-γ), that dephosphorylate CREB and reduce the Sp1 nuclear protein expression. This prevents their binding to the respective sites in the FN1 promoter and inhibits FN1 expression [185].

Several studies aimed at developing drugs targeting the β integrins to abrogate the FN1- β integrin signaling [184, 186], however we will not discuss this topic largely debated elsewhere.

FN1 has been related to therapy resistance in cancer and FN1 is one of the most altered gene related to cisplatin resistance [187]. Gao et al. identified mechanisms employed by FN1 to modulate the Wnt/β catenin pathway via its receptor β1 integrin, thus contributing to cisplatin resistance [188]. Gemcitabine, despite its application as a backbone of chemotherapy for pancreatic ductal adenocarcinoma [189], develops chemoresistance due to involvement of ECM-induced signals. Amrutkar and colleagues demonstrated that FN1 secreted by stromal pancreatic stellate cells promotes gemcitabine resistance in pancreatic cancer cells by activating ERK1/2. Furthermore, the use of FN1-blocking agents, such as a synthetic Arg-Gly-Asp-Ser (RGDS) peptide, abrogates the chemoresistance [190]. Moreover the role of FN1 in docetaxel resistance in NSCLC patients has been reported and FN1 expression inversely correlates to treatment response. Mechanistically, FN1 sustains the cell viability and proliferation and in parallel reduces the docetaxel-induced apoptosis inhibiting the caspase 8 [191]. Upon etoposide treatment, in the same lung cancer model FN1-β1 integrin signaling inhibits the chemotherapy-induced apoptosis due to a reduction of caspase 3 activity [192]. Small cell lung cancer (SCLC) has ECM enriched in FN1 as well as collagen IV and tenascin that reduce the apoptosis induced by multiple drugs, including doxorubicin, cyclophosphamide and etoposide. In this model the β1 integrin-dependent drug resistance is due to protein tyrosine kinase stimulation [193]. Chemotherapy but also radiotherapy resistance occurs in SCLC due to apoptosis inhibition following the FN1-β1 integrin signaling. In this regard it has been showed that lung cancer cell adhesion to FN1 modulates the cetuximab-dependent cytotoxicity and radiosensitation by the synthesis and secretion of FN1 and the activation of p38 MAPK/ATF2 pathway [194]. Similarly, the cell attachment to FN1 induces changes in NSCLC cells response to chemo- and radio-therapy. Cancer cells attached to FN1 show enhanced clusters of β1 integrin, which initiate its downstream signaling, upon treatment with radiotherapy, paclitaxel, mitomycin and cisplatin [195]. A peptide that inhibits the interactions of α5β1-integrin with FN1 increases the apoptotic responses of breast cancer cell lines to ionizing radiation [196]. Additionally, treatment with statins reduces melanoma cell adhesion to FN1 as well as to other matrix components, including collagens and laminin, in parallel to reduction of lung metastasis and cell invasion [197]. Exogenous FN1 confers Tamoxifen resistance in breast cancer through interaction with β1 integrin and modulates the activity of estrogen receptor [198] and nanoformulation, that simultaneously targets ER and FN1/β1 integrin interactions, has been proposed as potential therapeutic strategy for the management of breast tumors [199]. Mechanical changes promoted by FN1 folding and tumor stroma rigidity contribute to therapy cancer resistance. In human lung cancer cells unfolded type III domain of FN1 inhibited TRAIL induced apoptosis through the activation of a PI3K/Akt/αvβ5 signaling pathway [200].

Finally, FN1 is such a complex component of the ECM which relies with important implications in cancer therapy resistance in particular in lung cancer.

## Conclusions

In the last few years, thanks to the growing availability of advanced technologies integrating bioengineering, biophysics, biochemistry and imaging, the study of tumor matrisome has received great attention by researchers dedicated to the various fields of cancer biology. The fundamental role of ECM composition and architecture in the tumor development, progression and resistance to therapies is emerging as a promising tool for the definition of novel biomarkers useful in the stratification of patients to direct to the most appropriate therapy and for the identification of stroma-derived targets to design more efficacious treatments. As illustrated in this review, FN1 represents the driver of ECM organization and a structural scaffold thanks to its peculiar properties to self-assemble into fibrils. The implication of FN1 fibrillogenesis in the availability of growth factors and in particular of TGF-β1, strengthens its contribution in cancer progression, invasion and metastasis. FN1 expression and organization is altered in cancer mainly due to the pathological activity of CAFs, and in particular of specific CAF subtypes, that are emerging as significantly correlated with an immunosuppressive environment and associated with primary resistance to immunotherapies in patients with melanoma and NSCLC [132]. These novel implications in parallel to the concept that an abundant and rigid ECM, characteristic of tumor stroma represents a barrier for the infiltration of immune cells, will open new prospective in the study of FN1 also in the immunoncology field with a potential great impact in clinical practice.

## Data Availability

Not applicable.

## Abbreviations

BMDCs: Bone marrow-derived dendritic cell
CAFs: Cancer associated fibroblasts
CIP2A: Cancerous inhibitor of protein phosphatase 2A
cFN: Cellular fibronectin
DOC: Deoxycholate detergent
ECM: Extracellular matrix
EDA: Extra Domain A
EDB: Extra Domain B
EMT: Epithelial to mesenchymal transition
ERK2: Extracellular signal regulated kinase 2
EVs: Extracellular vesicles
FAK: Focal adhesion kinase
FN1: Fibronectin
FRET: Fluorescence resonance energy transfer
LTBP-1: Latent TGFβ-binding protein 1
LOX: Lysyl oxidase
MEFs: Mouse embryonic fibroblasts
MKL1: Megakaryoblastic leukemia-1
MMP: Metalloproteinase
MR: Magnetic resonance
NSCLC: Non-small cell lung cancer
pFN1: Plasma fibronectin
PI3K: Phosphoinositide 3-kinase
PPAR-γ: Peroxisome proliferator activated receptors
SFRP2: Secreted frizzled-related protein 2
SCLC: Small cell lung cancer
SPP1: Secreted phosphoprotein 1
SRF: Serum response factor
TβRII: Type II TGF-β receptor
TGF-β1: Transforming growth factor-β1
TLR: Toll-like receptor
TNF-α: Tumor necrosis factor α
TZDs: Thiazolidinediones
VEGFs: Vascular endothelial growth factors
